# The ability to return to work: a patient-centered outcome parameter following glioma surgery

**DOI:** 10.1007/s11060-020-03609-2

**Published:** 2020-09-22

**Authors:** Christian Senft, Marion Behrens, Irina Lortz, Katharina Wenger, Katharina Filipski, Volker Seifert, Marie-Thérèse Forster

**Affiliations:** 1 Department of Neurosurgery, Goethe-University Hospital, Schleusenweg 2-16, 60528 Frankfurt, Germany; 2 Department of Neurology, Goethe-University Hospital, Frankfurt, Germany; 3 Institute of Neuroradiology, Goethe-University Hospital, Frankfurt, Germany; 4 Neurological Institute (Edinger-Institute), Goethe-University, Frankfurt, Germany; 5 University Cancer Center Frankfurt – UCT, Frankfurt, Germany; 6 German Cancer Consortium (DKTK), Partner Site Frankfurt/Mainz, Heidelberg, Germany; 7 German Cancer Research Center (DKFZ), Heidelberg, Germany

**Keywords:** Return to work, Glioma, Brain tumor surgery, Quality of life

## Abstract

**Background:**

With refinements in diagnosis and therapy of gliomas, the importance of survival time as the sole outcome parameter has decreased, and patient-centered outcome parameters have gained interest. Pursuing a profession is an indispensable component of human happiness. The aim of this study was to analyze the professional outcomes besides their neuro-oncological and functional evaluation after surgery for gliomas in eloquent areas.

**Methods:**

We assessed neuro-oncological and functional outcomes of patients with gliomas WHO grades II and III undergoing surgery between 2012 and 2018. All patients underwent routine follow-up and adjuvant treatment. Treatment and survival parameters were collected prospectively. Repercussions of the disease on the patients’ professional status, socio-economic situation, and neurocognitive function were evaluated retrospectively with questionnaires.

**Results:**

We analyzed data of 58 patients with gliomas (WHO II: 9; III: 49). Median patient age was 35.8 years (range 21–63 years). Awake surgery techniques were applied in 32 patients (55.2%). Gross total and subtotal tumor resections were achieved in 33 (56.9%) and 17 (29.3%) patients, respectively, whereas in 8 patients (13.8%) resection had to remain partial. Most patients (n = 46; 79.3%) received adjuvant treatment. Median follow up was 43.8 months (range 11–82 months). After treatment 41 patients (70.7%) were able to resume a working life. Median time until returning to work was 8.0 months (range 0.2–22.0 months). To be younger than 40 at the time of the surgery was associated with a higher probability to return to work (p < .001). Multivariable regression analysis showed that patient age < 40 years as well as occupational group and self-reported fatigue were factors independently associated with the ability to return to work.

**Conclusion:**

The ability to resume professional activities following brain tumor surgery is an important patient-oriented outcome parameter. We found that the majority of patients with gliomas were able to return to work following surgical and adjuvant treatment. Preservation of neurological function is of utmost relevance for individual patients´ quality of life.

**Electronic supplementary material:**

The online version of this article (10.1007/s11060-020-03609-2) contains supplementary material, which is available to authorized users.

## Introduction

For decades, overall and progression-free survival have been the main outcome parameters for patients with gliomas. Gliomas are classified in Grades I–IV, according to morphological and genetic features that are laid out in the revised WHO classification of brain tumors [1]. Prognosis is dismal especially for patients with Grade IV tumors, even when administering multimodal treatment.

Today, personalized medicine approaches according to molecular and epigenetic tumor data tailor adjuvant treatment for patients with lower grade gliomas [2, 3]. With refinements in therapy and improvements in life expectancy, [4–6] the perception of glioma gradually shifts from a lethal to a chronic disease, with median survival times ranging between 5 and 15 years [6–9]. Therefore, effects of therapeutic interventions on patients’ quality of life have gained interest in recent years [10, 11].

Surgery plays a major role in the first-line treatment of gliomas [12–14]. Preserving neurological function is of upmost importance when it comes to surgical approaches and extent of tumor removal. Many studies have addressed surgical complications as well as sequelae of surgery on neurological outcome [15–17]. In contrast to brain tumor patients, more attention has been paid to the long-term health and well-being of people living longer with or having survived cancer [18, 19]. An emphasis has recently been placed to uphold also capabilities for social life, especially in lower-grade gliomas [11]. Latest reports also accentuate the importance of patient-reported outcome parameters [20].

Of particular concern in other cancers, but often overlooked in brain tumor patients due to traditionally poor survival, is the effect of the disease on productivity and work ability. For example, in breast cancer patients, the disruption of working life reportedly threatens not only their economic well-being, but also negatively affects their social relationships and personal satisfaction with life [21, 22].

Pursuing a professional career is an integral part of life and a major source of self-satisfaction and self-fulfillment. Diagnosis of a brain tumor severely disrupts also the professional lives of affected patients. Surgery and recovery from it, as well as any adjuvant treatment, may withhold patients from employment and thus their ability to continue normal lives. Side-effects of treatment or clinical deterioration from tumor progression might cause permanent inability to work. Few studies so far have specifically addressed return-to-work quota in brain tumor patients. Earlier studies focused on cognitive limitations in brain tumor patients, including patients suffering from benign tumors who returned to work, and found tasks requiring working memory highly challenging for these patients [23, 24].

So far, no clinical factors have been clearly established to answer a patient’s question, if and when they may return to work following surgery for glioma. Ng et al. recently presented details on 74 patients with WHO grade II gliomas only [25]. In their series, mean patient age was 35.7 years, and 66 patients (89.2%) were able to return to work. However, they did not find any factor associated with the ability to return to work.

With this report, we aimed to assess the impact of brain tumor surgery and adjuvant treatment on our patients’ occupational status as well as on their social and economic situation, and to identify clinical factors associated with their capability to return to work.

## Materials and methods

### Patients

We prospectively collected clinical data of patients with newly diagnosed gliomas between 8/2012 and 6/2018 who were scheduled to undergo surgery. All patients gave written consent prior to data collection. Patients who had biopsy only or were found to have WHO grade I or IV tumors were not included in this analysis. Detailed information on the occupational status and the time until patients resumed their professional careers, on their familial and economic situation and on quality-of-life-related aspects were obtained retrospectively by a one-time, detailed questionnaire well after adjuvant treatment.

For this analysis, we included only patients who were professionally active before tumor diagnosis. Patients were considered professionally active when they were employed, self-employed or unemployed but actively seeking for a job. If they were on permanent sick leave or retired, we considered them not professionally active, and these patients were not included. Our patients’ occupational status was assessed and grouped according to the International Standard Classification of Occupation, ISCO-08 [26]. This study was conducted with approval from our local ethics committee (SNO 04/09 and SNO 8/16).

### Postsurgical treatment and outcome measures

We looked at factors potentially influencing the ability of patients to resume their working lives, including age, WHO grade and histological subtypes of their respective tumor. Tumors were classified according to the WHO Classification of Tumours of the Central Nervous System 4th edition (2007) or revised 4th edition (2016), respectively, depending on disease onset. IDH mutational status was determined by immunohistochemistry as previously described [27]. Particularly, we assessed treatment interventions and their effect on the patients’ ability to return to work, such as the type or extent of surgery or the type of adjuvant treatment such as radiation and/or chemotherapy. At our institution, patients with tumors near speech-eloquent brain areas undergo awake surgery, whereas preservation of motor function during surgery is routinely assessed by transcortical monitoring and direct cortical or subcortical stimulation techniques in addition to measuring sensory evoked potentials as described previously [28, 29]. The extent of tumor resection was assessed by independent review of pre- and postoperative MRI and determined to be radiologically complete, subtotal (when there was less than 10% of the original volume as residual tumor), or partial, if less than 90% of the original tumor volume were removed. Pre- and postoperatively, an interdisciplinary tumor board gave treatment recommendations based on WHO grades and histological subtypes. All patients were followed up with clinical and radiological examinations in regular, usually three-monthly, intervals. Disease progression was defined according to the RANO criteria [30].

### Statistics

All analyses were performed with SPSS version 26 (IBM Inc., Armonk, New York). The association between dichotomized clinical and/or patient specific variables with the ability to return to work was assessed with chi-square or Fisher’s exact test, when appropriate. We performed a logistic regression analysis account for multivariable testing. The time until a professional life was resumed was analyzed with Kaplan–Meier estimates, for multivariate testing we used a cox regression analysis (backward stepwise). P-values < 0.05 were considered to be statistically significant.

## Results

For this analysis, 58 patients were included. Due to tumor location, 32 patients (55.2%) underwent tumor resection employing awake mapping and monitoring techniques to allow for intraoperative testing and preservation of speech function, in addition to motor or sensory evoked potential monitoring. All other patients underwent surgery under general anesthesia, employing motor or sensory evoked potential monitoring if deemed necessary according to tumor location. Early postoperative MRI revealed that complete or subtotal tumor removal could be achieved in 33 and 17 patients, respectively (56.9% and 29.3%, resp.), while a partial removal could only be achieved in 8 patients (13.8%). Baseline characteristics are provided in Table 1.

**Table 1:** Baseline characteristics

	N (%) or median [range]
Gender	
Female	27 (46.6)
Male	31 (53.4)
Age (years)	35.8 [21.8–63.6]
Preoperative KPS	100 [80–100]
Preoperative seizures	
Yes	41 (70.7)
No	17 (29.3)
Tumor localization	
Frontal	35 (60.3)
Temporal	15 (25.9)
Parietal	7 (12.1)
Occipital	1 (1.7)
Histopathological diagnosis	
Grade II	
Astrocytoma	4 (6.9)
Oligodendroglioma	4 (6.9)
Xanthoastrocytoma	1 (1.7)
Grade III	
Astrocytoma	31 (53.4)
Oligodendroglioma	17 (29.3)
Xanthoastrocytoma	1 (1.7)
IDH mutation	
Yes	47 (81)
No	11 (19)
Extent of resection	
Gross total resection	33 (56.9)
Subtotal resection	17 (29.3)
Partial resection	8 (13.8)
Marital status	
Married/cohabiting	47 (81.0)
Single/divorced	12 (20.7)

*KPS* Karnofsky Performance Score, *IDH* isocitrate dehydrogenase

Following tumor board recommendation and patients’ personal preference, most patients (n = 46, 79.3%) received adjuvant treatment**.** Chemotherapy or radiation therapy only were administered in 3 patients (5.2%). 43 patients (74.1%) received both, chemotherapy and radiation therapy. 12 patients (20.7%) were treated only surgically, without adjuvant treatment. Before treatment, 41 (70.7%) patients had seizures, whereas 17 (29.3%) did not.

**Fig. 1:**
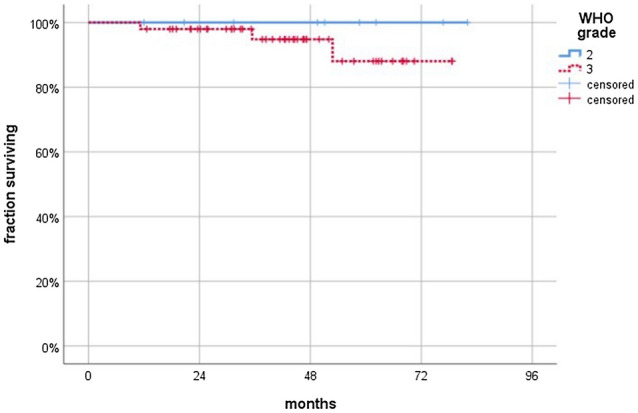
Kaplan Meier graph showing patient survival stratified by WHO grade. Median survival was not reached

Median follow-up time of all patients was 44.3 months (range 11–82 months). During follow-up, 10 patients (17.2%) showed disease progression and 3 patients have died. Figure 1 depicts Kaplan–Meier curves stratified by WHO grade. Median survival was not reached.

### Return to work

Following surgery and adjuvant treatment, if applicable, of the 58 patients, 41 (70.7%) were able to return to work, whereas 17 (29.3%) were not; of the latter, 2 patients retired, and 15 patients were on permanent sick leave, including the 3 patients who had died.

When given the questionnaires, the majority of patients (n = 28, 48.3%) reported that their economic status was unchanged at follow-up. However, a large proportion of patients (n = 16, 27.6%) reported a reduction in income, whereas a minority (n = 6, 10.3%) had an increase in income. 5 patients (8.6%) were not willing to share economic information, and data were not available for the patients who had died.

The most prevailing self-reported symptom was fatigue (31 patients out of 51 reports, 60.8%). Half of the patients (25 out of 50 reports) reported memory disturbances, while a minority described difficulties concentrating (22 patients out of 51 reports, 43.1%) or finding words (18 patients out of 51 reports, 35.3%).

At the time of analysis, 32 (55.2%) patients were on antiepileptic drug treatment, but only 6 (10.3%) patients were still experiencing seizures. To treat epilepsy, 19 (32.8%) patients received a monotherapy with levetiracetam and 12 (20.7%) had a combination therapy that included levetiracetam. Details on clinical and socio-economic data at follow-up are given in Table 2.

**Table 2:** Survival, clinical and sociodemographic outcomes at follow-up

Outcomes	N (%) or Median [range]
Overall follow-up period, months	43.9 [11.2–82.0]
Overall survival	
Alive at last follow-up	55 (94.8)
Oncological outcome^a^	
Progressive disease	7 (12.7)
Stable disease	48 (87.3)
Clinical outcome^a^	
KPS	100 [60–100]
Seizures	64 (7.3)
Anticonvulsive therapy	29 (52.7)
Levetiracetam	19 (34.5)
Levetiracetam + 1 other AED	10 (18.2)
Marital status^a^	
Married/cohabiting	39 (70.9)
Single/divorced	16 (29.1)
Economic status^a^	
Increased income	6 (10.9)
Decreased income	16 (29.1)
Income unchanged	28 (50.9)
Not available	5 (9.1)

*KPS* Karnofsky performance scale, *AED* antiepileptic drug

^a^Excluding deceased patients

#### Assessment of factors associated with resumption of professional activities

When stratifying between patients younger than 40 years of age and those 40 years and above, younger patients were more likely to resume professional activities than older patients (94.1% vs. 37.5%, Table 3). Patient age was statistically significantly associated with the ability to return to work (P < 0.001, Fisher’s exact test).

**Table 3:** Factors associated with return to work according to monovariable analysis

Factor	Return to work		Total	P
	Yes	No		
Age				
< 40 years	32 (94.1%)	2 (5.9%)	34	**0.001**^#^
≥ 40 years	9 (37.5%)	15 (62.5%)	24	
Extent of resection				
Complete/subtotal	37 (75.5%)	12 (24.5%)	49	0.106^#^
Partial	4 (24.4%)	5 (55.6%)	9	
ISCO-08				
Groups 1–2	23 (85.2%)	4 (14.8%)	27	**0.041**^#^
Groups 3–9	18 (58.1%)	13 (41.9%)	31	
Fatigue^a^				
Yes	20 (64.5%)	11 (35.5%)	31	**0.017**^#^
No	19 (95.0%)	1 (5.0%)	20	
Epilepsy^a^				
Controlled	39 (75.0%)	13 (25.0%)	52	0.055^#^
Having seizures	2 (33.3%)	4 (66.7%)	6	

P values < 0.05 were considered statistically significant

*ISCO-08* international standard classifications of occupations (ref. no [26])

^#^Fisher’s exact test

^a^At follow-up

Also, occupational status as determined according to ISCO-08 was associated with return to work. Patients within groups 1–2 were more likely to return to work than patients in groups 3–9 (proportion returning to work 85% vs. 58%; P = 0.041, Fisher’s exact test; Table 4).

**Table 4:** Details on occupational groups and their association with return to work

ISCO-08 Groups	At presentation	At follow-up		
			RTW, n (%)	
	Professionally active patients, n	Patients alive and not retired^†^, n	Yes, n (%)	No, n (%)
Group 1—Managers	4	4	3 (75)	1 (25)
Group 2—Professionals	22	21	20 (95.2)	1 (4.8)
Group 3—Technicians and Associate Professionals	12	10	8 (80)	2 (20)
Group 4—Clerical Support Workers	4	4	4 (100)	0
Group 5—Services and Sales Workers	7	7	2 (28.6)	5 (71.4)
Group 7—Craft and Related Trads Workers	6	6	3 (50)	3 (50)
Group 9—Elementary Occupations	3	1	1 (100)	0
	58	53	41 (77.4)	12 (22.6)

^†^3 patients have died, 2 have retired

*ISCO-08* International standard classification of occupations (ref. no [26]), *RTW* return to work

There was a trend for a higher chance of patients returning to work after more extensive resections (removal of 90% or more of the preoperative tumor volume) compared to a less extensive resection (proportion returning to work 76% vs. 44%; P = 0.106, Fisher’s exact test, Table 3).

While the proportion of patients reporting fatigue was higher in the subgroup of patients who did not return to work (95% vs. 64% in the subgroup who did return to work; P = 0.017, Fisher’s exact test; Table 3), there were no significant differences between groups regarding other symptoms (supplementary data).

Interestingly, we found that the proportion of patients still experiencing seizures despite antiepileptic drug treatment tended to be higher in the subgroup of patients who did not return to work compared to those who did (30.8% vs. 5.1%; P = 0.055, Fisher’s exact test).

Contrastingly, gender, WHO grade, KPS score at presentation, tumor histology, IDH mutational status, or administration of adjuvant therapy were not found to be statistically significantly associated with the ability of patients to return to work. Likewise, patients’ marital status or the presence of epilepsy at diagnosis was not associated with the ability to return to work (supplementary data).

#### Logistic regression analysis

We performed a multinomial logistic regression analysis to account for multivariable testing. We included all factors that showed an association according to monovariable analysis. Accordingly, age (P < 0.02), ISCO groups (P < 0.02), and fatigue (P < 0.03) were independently associated with the ability to return to work, while extent of resection (P = 0.38) and uncontrolled seizures (P = 0.18) were not.

### Duration of absence from work

**Fig. 2:**
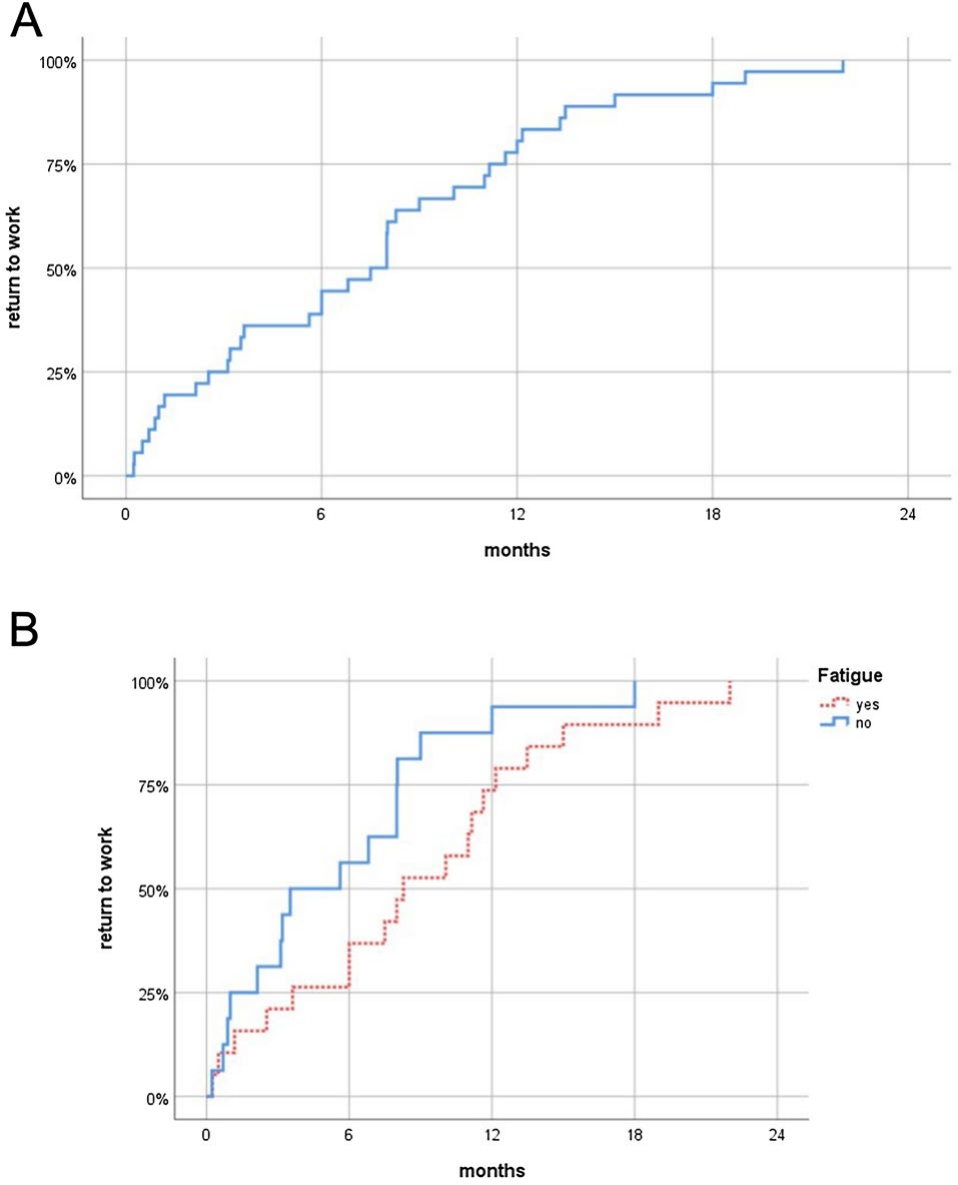
Kaplan Meier graphs showing the duration until return to work for the entire cohort (**a**) and stratified by fatigue (**b**) as reported at follow-up. There was a statistical trend for the differences between patients with or without fatigue to be significant (P < 0.067, Log-rank test)

Data could be established for 36 out of 41 patients returning to work. We then performed Kaplan–Meier analyses to identify factors potentially associated with an early return to work. The median duration until return to work was 7.5 months (95%-confidence interval [CI] 5.1–9.9 months; Fig. 2). One patient resumed work as early as 7 days following surgery; the longest interval between surgery and resumption of work was 22.0 months.

There were no statistically significant differences in the time until patients returned to work when stratifying according to gender, age, tumor location, type of surgery, extent of resection, tumor histology, WHO grade, IDH mutation, adjuvant radiotherapy, presence of epilepsy at presentation or during follow-up, marital status, or ISCO groups (data not shown; P > 0.1 for all, Log-rank test).

When looking at self-reported symptoms, there was a trend for fatigue only to be associated with the time until professional activities were resumed: 19 patients reporting fatigue returned to work later than 16 patients who did not (median time until return to work: 8.3 vs. 3.5 months; P = 0.067, Log-rank test; Fig. 2). There were no associations between return to work times and word-finding difficulties, memory disturbances, or difficulties concentrating (P > 0.2 for all, Log-rank test).

Likewise, there was a trend for 26 patients receiving chemotherapy to return to work later than 10 patients who did not (median time until return to work: 8.0 vs. 5.6 months; P = 0.058, Log-rank test). The administration of radiation therapy did not affect the duration until return to work on a statistically significant level (P = 0.11, Log-rank test).

#### Cox-regression analysis

Following a multivariable Cox-regression analysis, the association between chemotherapy on return to work times did not remain independently significant (P = 0.18), while the trend for fatigue persisted (P = 0.076).

## Discussion

We observed that most of our patients were able to resume their working lives following brain tumor surgery.

In contrast to the recent study from Ng et al. [25] we also included patients with WHO grade III gliomas, and the proportion of patients receiving adjuvant treatment was greater in our cohort. Importantly, we could establish younger age and higher occupational status variables to be statistically significantly associated with the ability to return to work following multivariate analyses. We also observed that the proportion of patients experiencing fatigue was significantly higher in the subgroup of patients who did not return to work, and fatigue was associated with a longer interval until professional activities could be resumed.

A multitude of literature suggests that employing awake mapping and monitoring techniques is particularly beneficial for glioma patients [16, 17, 31–33]. We frequently use awake surgery for tumor resection, and in our current series, more than half of the patients underwent awake craniotomy. Other groups have argued that awake surgery may facilitate return to work [33–35], most likely because of the high chance of preserving neurological function. While in general glioma surgery may result in a survival benefit [36, 37] or relief from tumor symptoms [38], our results did not show an effect of extent of resection on the ability or duration of patients returning to work.

Likewise, compared to general anesthesia with cortical and subcortical stimulation for MEP monitoring and continuous SSEP recording, employing awake techniques in our series did not influence the ability to return to work or the time until resumption of work on a statistically significant level. Tumor location clearly contributes to the decision to perform awake or non-awake surgery, but we did not find tumor location to be associated with return to work.

Radiation therapy is known to negatively influence cognitive function, and this effect may occur as a late sequel of therapy [39]. Interestingly, we did not observe any adverse effect of radiation therapy in terms of the patients´ ability to return to work. In contrast, patients who received chemotherapy tended to suspend professional activities for a longer period of time. Adjuvant therapy, however, did not influence the general capability of patients to return to professional lives.

Our results compare well with previously published literature. Altshuler et al. studied the association of genetic alterations with neurocognitive function and ability to return to work in a series of 34 patients with gliomas WHO grades II and III who did not receive adjuvant treatment following surgery [40]. Here, 42% of the patients returned to work within three months following surgery. In our current series, this proportion was only slightly lower with 4 out of 12 patients (33.3%) without adjuvant treatment having returned to work three months after surgery (data not shown).

The results in patients with lower grade gliomas juxtapose with observations made in patients with higher grade tumors. Starnoni et al. reported that fewer than 20% of patients with glioblastoma were able to return to work [41]. Very recently Yoshida et al. reported on 50 patients undergoing awake surgery for gliomas of WHO grades II–IV [35]. In their series, only 54% of patients were able to return to work. We did not observe an association between WHO grade and the ability to return to work, but the duration of absence from work was longer for patients with WHO grade III tumors compared to WHO grades II, albeit this difference was not significant (8.0 vs. 5.6 months, data not shown).

Median age of patients harboring lower grade gliomas is below 50 years, and a vast majority of patients are employed when being confronted with tumor diagnosis. Aside from receiving life-prolonging treatment, being able to generate income and sustain their lives independently is essential for patients’ well-being and happiness [42, 43]. Of note, we found that patients with higher occupational status, i.e. groups 1 and 2 according to ISCO-08, were more likely to return to work than patients with less elaborate professions which usually are lower paid for. We can only speculate as to how this can be explained, but for these groups their workplaces might provide greater opportunities for self-fulfillment in addition to above-average salaries.

Our study is certainly limited by the fact that we were not able to determine whether patients who did not return to work made that decision due to disease related factors or personal choice. Another limitation is the fact that a structured assessment of working status was not repeatedly performed during the regular follow-up, but only once.

Due to legal or socio-cultural differences [44], the results observed in our study might very well be different from other regions of the world. In a recent study from Japan for example, being the sole breadwinner in contrast to patient age was a predictive factor for returning to work according to multivariate analysis [35]. In our cohort, marital status did not seem to play a role. With our social security system being very supportive, motivating pressures to return to work may be lower in our country compared to others where financial insecurity is higher.

The ability of patients to maintain or return to professional activities after treatment is influenced by multiple intrinsic and extrinsic factors. Thus, variables that affect patients’ capability or motivation to return to work may be identified only in larger cohorts. We did, however, observe that fatigue as a frequently encountered burden of gliomas is of great importance when it comes to patients’ ability to return to work following glioma surgery and adjuvant treatment. Practitioners should emphasize this aspect, as other factors like age or occupational status cannot be therapeutically addressed.

We conclude that the ability to return to work is an important patient-centered outcome parameter in glioma treatment that should be evaluated more systematically.

## Electronic supplementary material

Below is the link to the electronic supplementary material.

Supplementary file1 (DOCX 19 kb)
