# Supplementary material for: The ability to return to work: a patient-centered outcome parameter following glioma surgery
Source: J Neurooncol. 2020 Sep 22;149(3):403–11. doi: 10.1007/s11060-020-03609-2 (PMC7609423; doi:10.1007/s11060-020-03609-2)
Supplement: Supplementary file 1 — Supplementary file1 (DOCX 19 kb) [file 11060_2020_3609_MOESM1_ESM.docx]

**Association of patient characteristics and the ability to return to work**

| **Return to work** | **Yes** | | | | **No** | **Total** |  | |
| --- | --- | --- | --- | --- | --- | --- | --- | --- |
| **Gender** |  | | | |  |  |  | |
| Male | 23 (74.2%) | | | | 8 (25.8%) | 31 | P=.53 ^†^ |  |
| Female | 18 (66.7%) | | | | 9 (33.3%) | 27 |  | |
| **Marital status** |  | | | |  |  |  | |
| Cohabitating / married | 27 (64.3%) | | | | 15 (35.7%) | 42 | P=.112 ^#^ | |
| Single / divorced | 14 (87.5%) | | | | 2 (12.5%) | 16 |  | |
| **WHO Grade** |  | | | |  |  |  | |
| °II | 8 (88.9%) | | | | 1 (11.1%) | 9 | P=.258 ^#^ | |
| °III | 33 (67.3%) | | | | 16 (32.7%) | 49 |  | |
| **Tumor histology** |  | | | |  |  |  | |
| Astrocytoma | 25 (71.4%) | | | | 10 (28.6%) | 35 | P=.606 ^†^ | |
| Oligodendroglioma | 14 (66.7%) | | | | 7 (33.3%) | 21 |  | |
| Other | 2 (100%) | | | | 0 (0.0%) | 2 |  | |
| **IDH1_R132H mutational status** | | | | |  |  |  | |
| IDH1 mutation + | 35 (74.5%) | | | | 12 (25.5%) | 47 | P=.191 ^†^ | |
| No IDH1 mutation | 6 (54.5%) | | | | 5 (45.5%) | 11 |  | |
| **Epilepsy at presentation** | | | |  |  |  |  | |
| Seizures | 28 (68.3%) | | | | 13 (31.7%) | 41 | P=.753 ^#^ | |
| No seizures | 13 (76.5%) | | | | 4 (23.5%) | 17 |  | |
| **Adjuvant treatment** |  | | | |  |  |  | |
| Adjuvant treatment | 31 (67.4%) | | | | 15 (32.6%) | 46 | P=.478 ^#^ | |
| No adjuvant treatment | 10 (83.3%) | | | | 2 (16.7%) | 12 |  | |
| **Word finding disorders^*^** | | |  | |  |  |  | |
| Yes | 13 (72.2%) | | | | 5 (27.8%) | 18 | P=.597 ^†^ | |
| No | 26 (78.8%) | | | | 7 (21.2%) | 33 |  | |
| **Difficulty concentrating^*^** | |  | | |  |  |  | |
| Yes | 15 (68.2%) | | | | 7 (31.8%) | 22 | P=.224 ^†^ | |
| No | 24 (82.8%) | | | | 5 (17.2%) | 29 |  | |
| **Memory problems^*^** |  | | | |  |  |  | |
| Yes | 19 (76.0%) | | | | 6 (24.0%) | 25 | P=.733 ^†^ | |
| No | 20 (80.0%) | | | | 5 (20.0%) | 25 |  | |

**^*^**  at follow-up; ^#^ Fisher’s exact test, ^†^ Pearson χ2 test
